# Vision Fine‐Tunes Predictions of Bimanual Self‐Touch

**DOI:** 10.1111/ejn.70435

**Published:** 2026-02-22

**Authors:** Noa Cemeljic, Konstantina Kilteni

**Affiliations:** ^1^ Department of Neuroscience Karolinska Institutet Stockholm Sweden; ^2^ Donders Institute for Brain, Cognition and Behaviour Radboud University Nijmegen the Netherlands

**Keywords:** internal forward models, self‐touch attenuation, somatosensory modulation, vision

## Abstract

Self‐touch is attenuated compared to external touch due to internal forward models predicting the somatosensory consequences of our movements. These self‐touch predictions are continuously updated during the movement using the available sensory input, resulting in a precise temporal tuning of somatosensory perception. However, the contributions of different sensory inputs, such as vision, to the predictions of the forward models and thus the resulting modulation of somatosensory perception remain unknown. In this pre‐registered study, participants discriminated forces applied to their left index or ring finger during a reaching movement of the right hand towards the left hand, performed both with and without visual input. When vision was available, somatosensory perception was gradually attenuated during the movement and peaked at the time of self‐touch, replicating our previous findings. Without visual input, this temporal tuning was reduced, as somatosensory perception was more uniformly, rather than gradually, attenuated throughout the movement. Our findings thus indicate that vision increases the precision of self‐touch predictions, thereby fine‐tuning the temporal modulation of somatosensory perception during movements to self‐touch.

## Graphical abstract

When we move to touch ourselves, our somatosensory perception is gradually attenuated due to the predictions of the internal forward models about the somatosensory consequences of our movements. Here, we show that visual input increases the precision of these self‐touch predictions, leading to a more pronounced temporal tuning of somatosensory attenuation during movements to self‐touch.

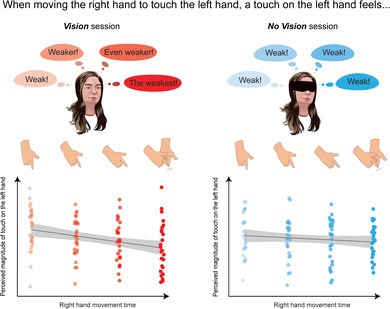

**Abbreviations**
ANOVA: analysis of variance
FDR: false discovery rate
IQR: interquartile range
JND: just noticeable difference
PSE: point of subjective equality
SD: standard deviation
SEM: standard error of the mean
TTL: transistor‐transistor logic

## 1 Introduction

Imagine lying in your bed when you suddenly feel an itchy sensation on your left hand. ‘Ugh… that mosquito again’, you think, and with your eyes still closed, you reach over with your right hand to swat it away. Soon, the same sensation returns—this time, you open your eyes and repeat the movement. In both cases, whether guided by sight or not, your brain accurately anticipates the touch sensation caused by your own movement, but not that of the mosquito. So, how does the brain predict the sensory consequences of our movements, both when we can see our body and when we cannot?

Motor control theories posit that the brain predicts self‐generated stimuli through an internal forward model (Franklin and Wolpert 2011; Jordan and Rumelhart 1992; McNamee and Wolpert 2019; Miall and Wolpert 1996), likely implemented in the cerebellum (Krakauer and Mazzoni 2011; Shadmehr and Krakauer 2008; Welniarz et al. 2021; Wolpert et al. 1998). For example, when we reach with the right hand (the effector) to touch our left hand (the target), the forward model uses a copy of the right hand's motor command—the efference copy—to predict the future state of the moving limb and the associated somatosensory consequences of the movement, including the contact between the moving right hand and the static left hand (i.e., self‐touch) (Bays and Wolpert 2007, 2008; Blakemore et al. 2000; Kilteni 2023, 2025; Wolpert and Flanagan 2001). These predictions manifest as attenuated perceptual responses and attenuated neural activity following self‐touch compared to externally generated touch of identical intensity—the self‐touch/pressure feels weaker or less ticklish (Asimakidou et al. 2022; Bays et al. 2005, 2006; Blakemore, Frith, and Wolpert 1999; Cemeljic et al. 2025; Job and Kilteni 2023; Kilteni et al. 2018, 2019, 2020, 2021; Kilteni and Ehrsson 2017a, 2017b, 2022; Lalouni et al. 2021, 2025; Shergill et al. 2003; Timar et al. 2023; Valè et al. 2025; Weiskrantz et al. 1971; Wolpe et al. 2016, 2018) and elicits weaker responses in the somatosensory cortices and cerebellum (Blakemore et al. 1998, 2001; Blakemore, Wolpert, and Frith 1999; Hesse et al. 2010; Job et al. 2025; Kilteni et al. 2023; Kilteni and Ehrsson 2020, 2024; Shergill et al. 2013).

We recently demonstrated a gradual perceptual attenuation of somatosensory input during visually guided movements to self‐touch (Cemeljic et al. 2025). Specifically, participants perceived touches applied to their left hand as gradually weaker in intensity as the right hand reached to make contact, and maximal attenuation was observed at the moment the two hands made the predicted contact. This temporal tuning during the reaching may reflect the continuous updating of forward model predictions as the new sensory input from the moving limb (i.e., new visual and proprioceptive input) is received during the ongoing movement (Brenner and Smeets 2018; Shadmehr et al. 2010; Shadmehr and Krakauer 2008) and used to refine the predicted time of self‐touch. For example, seeing the moving right hand approaching the target left hand, along with feeling the updated limb posture, provides input to the forward model to make more accurate predictions about when the contact between the two hands will occur. In other words, as the movement progresses, the probability of self‐touch timing increases based on the most recent sensory information, leading to gradually increasing attenuation of somatosensory perception.

Building on these earlier findings, the present study aimed to investigate the influence of vision on the temporal modulation of somatosensory perception during movements to self‐touch. Earlier evidence in sighted adults has shown that vision is crucial for maintaining movement accuracy (Desmurget et al. 1997; Elliott et al. 1991; Holmes and Spence 2005; Prablanc et al. 1979; Rossetti et al. 1995; Woodworth 1899) and for enabling rapid movement correction (Franklin and Wolpert 2008; Hansen et al. 2008; Reichenbach et al. 2009; Sarlegna et al. 2003, 2004; Saunders and Knill 2004; Tremblay et al. 2017). Visual signals are thus thought to play an important role in forming and updating the forward model predictions, ensuring successful motor control (Bernier et al. 2006; Brenner et al. 2023; Jayasinghe et al. 2021; Shadmehr and Krakauer 2008; Sober and Sabes 2003). We therefore reasoned that vision would also contribute to the formation and updating of predictions regarding the timing of self‐touch, and thus to the temporal tuning of somatosensory perception during the movement. In the present pre‐registered study (https://osf.io/z2wju), participants reached with their right hand towards their left hand both when visual input was available and when blindfolded. During the reaching movement of the right hand, we probed their somatosensory perception on the passive left hand at various time points. We hypothesized that the gradual attenuation of somatosensory perception observed during visually guided movements to self‐touch (Cemeljic et al. 2025) may vary depending on whether participants had access to visual input to refine their self‐touch predictions.

## 2 Results

**FIGURE 1:**
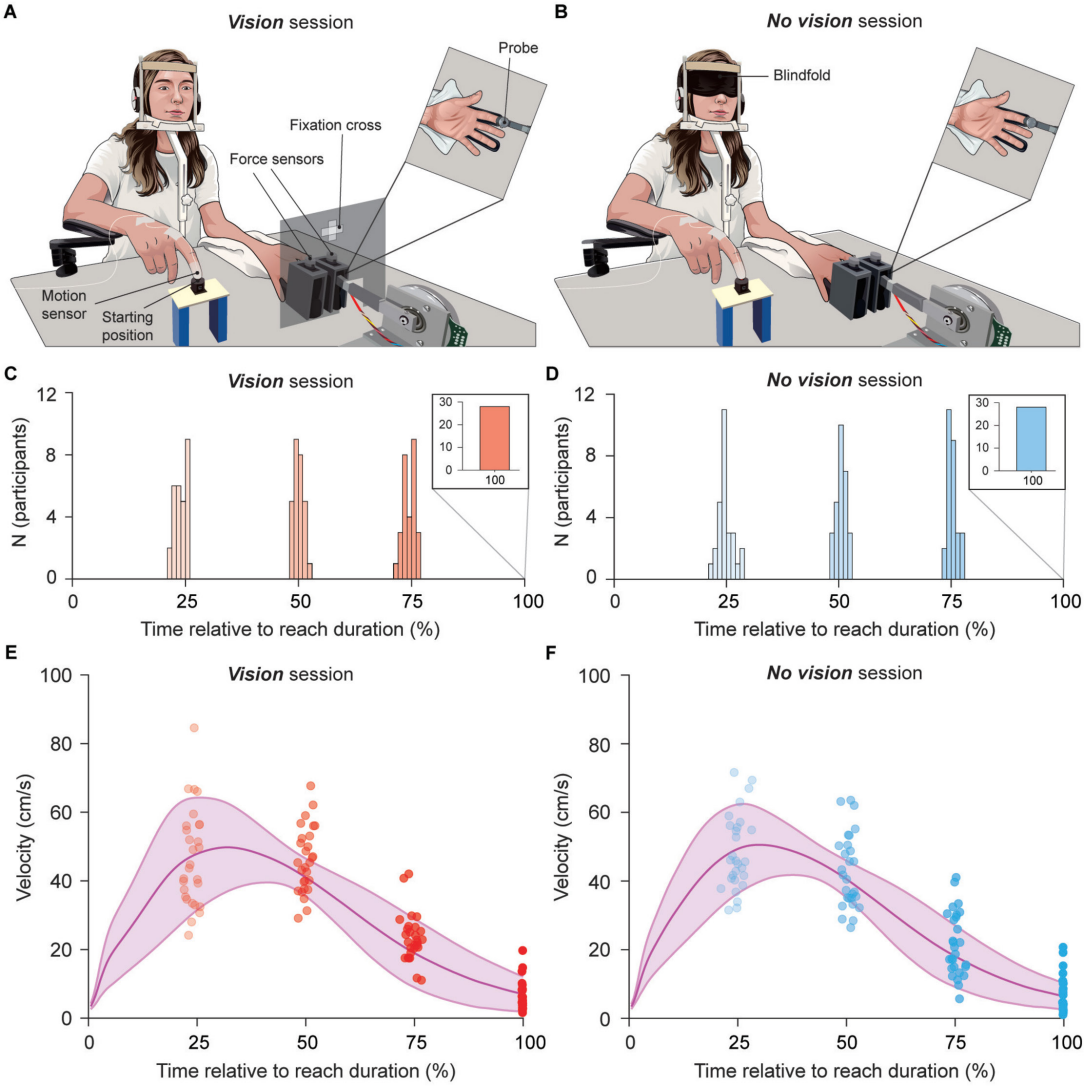
Experimental methods, design and velocities of the reaching movements. (A,B) Participants made a reaching movement with their right hand from the *starting position* towards one of the *force sensors* placed above their left index or ring finger, concluding the movement with a tap on the sensor with their right index finger. During the *baseline* block, the participants kept the right index finger still at the *starting position*. The *motion sensor* attached to the right index finger tracked participants' movements in every trial. Participants rested their right elbow and forearm on the elbow pad, and a chin rest was provided for head support. The tactile stimuli were delivered through the *probe* attached to the motor. In every trial, the probe delivered two brief forces on the pulp of the participants' left index or ring finger. In all trials of both sessions, participants kept their left hand relaxed inside a vacuum pillow. Half of the participants always received the forces on their left index finger, and the other half on their ring finger—only the index finger is shown here for illustration purposes. Participants completed the same task in both *Vision* and (A) *No vision* (B) sessions, with the only difference being the use of a *blindfold* in the *No vision* session. In the *Vision* session, participants were asked to focus their eyes on the *fixation cross* positioned above their left hand. For illustration purposes, the barrier with the fixation cross appears transparent, but it was opaque during the experiment. Participants also wore headphones through which white noise and reaching instructions were delivered. (C,D) Histograms showing the distribution of average times across participants when the *test* force was applied in *early*_25*%*_ (25% opacity), *mid*_50*%*_ (50% opacity), *late*_75*%*_ (75% opacity) and *target* (100% opacity) trials in *Vision* (C) and *No vision* (D) sessions. Times are expressed as percentages of the reaching movement duration and binned into 1% intervals. In *target* trials, the *test* force was delivered simultaneously with the end of the movement, *i.e.,* with the tap on the force sensor. (E,F) Average velocity profiles in *Vision* (E) and *No vision* (F) sessions, overlaid with individual average times and velocities when the *test* force was delivered in *early*_25*%*_ (25% opacity), *mid*_50*%*_ (50% opacity), *late*_75*%*_ (75% opacity) and *target* (100% opacity) trials. The shaded area represents the standard deviation. Although the 3D velocity of each participant was used for analysis, only the rectified velocity along the horizontal axis between the two hands, averaged across all participants, is shown here for illustration purposes.

Twenty‐eight participants (15 female, 27 right‐handed and one ambidextrous, aged 18–35 years) completed two experimental sessions in a counterbalanced order: the *Vision* session (Figure 1A) and the *No vision* session (Figure 1B). The procedure was identical between the two sessions, except that participants were blindfolded for the entire duration of the *No vision* session (Figure 1B, *blindfold*). Participants' left hands were positioned palm‐up, with two force sensors placed above the left index and ring fingers (Figure 1A, *force sensors*). At the start of each trial, participants reached with their right hand towards either their left index or ring finger and concluded the movement by tapping the force sensor above the instructed finger. Participants' movements were tracked using a motion sensor attached to their right index finger (Figure 1A, *motion sensor*).

Using an established force discrimination task (Asimakidou et al. 2022; Bays et al. 2005, 2006; Cemeljic et al. 2025; Job and Kilteni 2023; Kilteni 2023; Kilteni et al. 2019, 2020, 2021, 2023; Kilteni and Ehrsson 2022, 2024; Timar et al. 2023; Xiong et al. 2025), we quantified the perceived intensity of forces applied to the participant's left hand via a motor‐controlled probe (Figure 1A, *probe*). Two forces, the *test* and the *comparison* force, were applied in every trial, and half of the participants always received the two forces on their left index finger, whereas the other half always received forces on their left ring finger. The *test* force was always 2 N in intensity, while the *comparison* force had variable intensity between 1 and 3 N.

There were two blocks of trials. In the *baseline* block, both forces were delivered while the participants' hands were at rest (*baseline* trials). In the *reaching* block, the *test* force was delivered at one of several time points during the reaching movement: specifically, at 25%, 50% or 75% of the movement time (*early*_25%_, *mid*_50%_ and *late*_75%_ trial groups) or simultaneously with the participant tapping the force sensor above the instructed finger (*target* trials) (Figure 1C,D). The *comparison* force was always delivered after the reaching movement was concluded. At the end of each trial, participants responded as to which of the two forces (i.e., the first/*test* or the second/*comparison* force) felt stronger.

### 2.1 Comparable Movement Execution Between *Vision* and *No Vision* Sessions

We first compared the participants' movements between the *Vision* and *No vision* sessions. We reasoned that if participants executed comparable movements in both sessions, any observed perceptual effects could be attributable to the availability of vision, rather than to differences in movement execution. To ensure this, we trained each participant prior to the experiment to produce similar reaching movements with comparable velocity and duration across the *Vision* and *No vision* sessions. Indeed, the movement durations, peak velocities and the forces they applied on the sensors did not differ significantly between the two sessions (Figures 1E,F and S1 and Text S1). A Bayesian analysis further supported the absence of differences (all *BF*_01_ > 3) (Text S1).

### 2.2 Comparable Overall Attenuation of Forces Between *Vision* and *No Vision* Sessions

Next, we fitted the participants' responses with a psychometric curve for each trial group (*early*_25*%*_, *mid*_50*%*_, *late*_75*%*_ and *target*). From the fits, we extracted the point of subjective equality (PSE), which represents the perceived intensity of the *test* force (2 N). Because we observed no differences in the PSEs based on whether participants pressed above the same finger where they received the stimulus or not (Text S2 and Figure S2), we pooled the responses across both press locations for each trial group and fitted the pooled data. All psychometric model fits of the pooled data (Figures S3–S4) were considered very good (McFadden's *R* ^2^ > 0.38).

**FIGURE 2:**
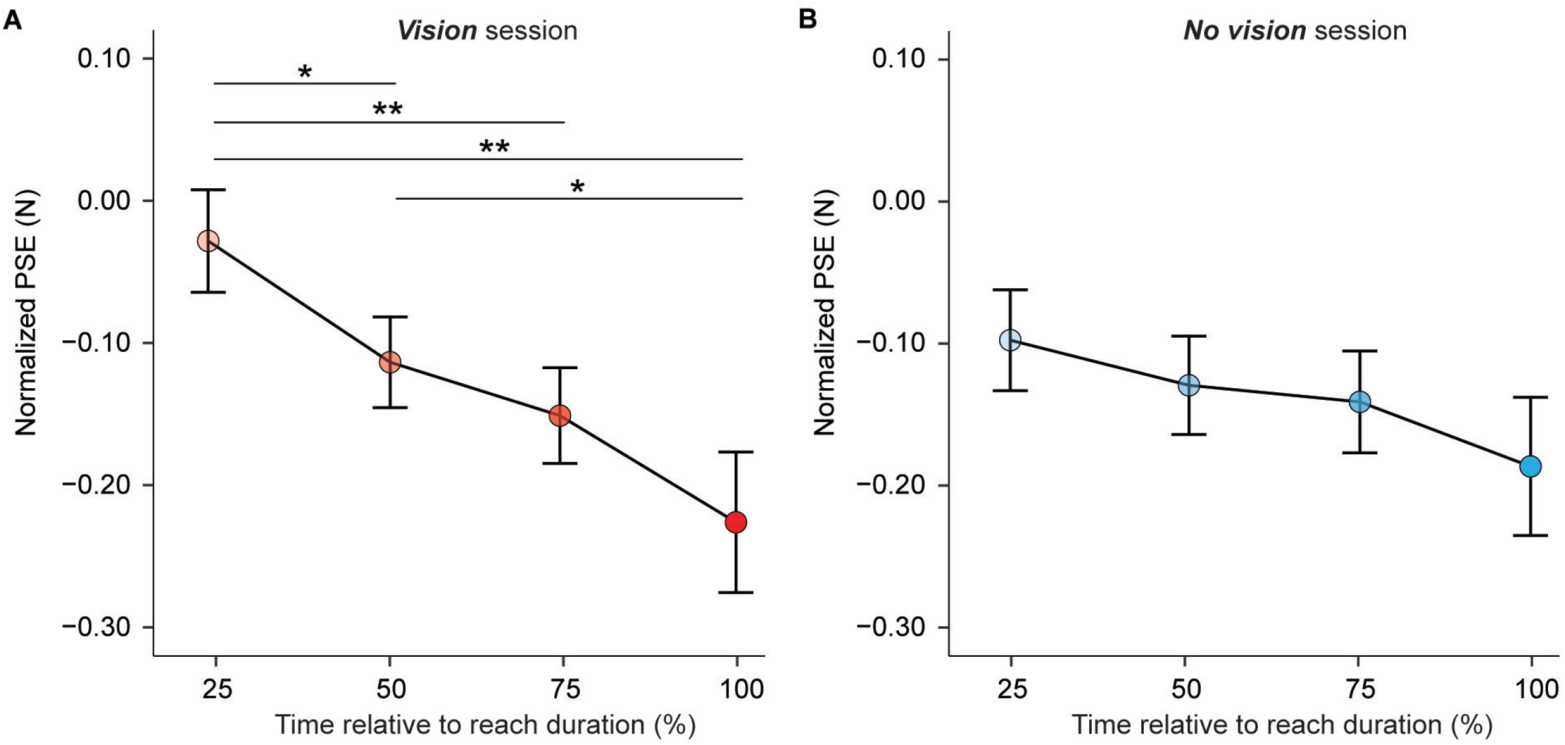
Baseline‐normalized PSE values in *Vision* and *No vision* sessions. (A,B) Baseline‐normalized group PSE values for each trial group (mean ± SEM) in *Vision* (A) and *No vision* (B) sessions. (A) In the *Vision* session, the touch felt significantly weaker as the reaching movement progressed, reaching its minimum at the time of contact between the hands (*target* trials). Asterisks show significant pairwise comparisons after FDR correction for multiple comparisons (*p < 0.05, **p < 0.01). (B) In the *No vision* session, there were no significant differences between trial groups, indicating the absence of temporal tuning of somatosensory perception during the reaching movement.

Following our pre‐registered analysis, we next compared the normalized PSEs (PSE_reaching_—PSE_baseline_) within each experimental session (*Vision* and *No vision*), treating the trial group (*early*_25*%*_, *mid*_50*%*_, *late*_75*%*_, *target*) as a categorical variable. In the *Vision* session, the ANOVA revealed a significant main effect of the trial group (*F*[1.902, 51.355] = 7.606, *p* = 0.002, *η*_*p*_^2^ = 0.220). Replicating our earlier findings (Cemeljic et al. 2025), post hoc comparisons showed that PSEs were decreasing as the *test* force was delivered closer to the predicted time of self‐touch. Specifically, PSEs were significantly lower in the *mid*_50*%*_ trials compared with the *early*_25*%*_ trials (*n* = 28, *t*(27) = −3.001, *p* = 0.011 *FDR corrected*, CI^95^ = [−0.144, −0.027], *d* = −0.567), the *late*_75*%*_ trials compared with the *early*_25*%*_ trials (*n* = 28, *t*(27) = −4.003, *p* = 0.001 *FDR corrected*, CI^95^ = [−0.186, −0.060], *d* = −0.756), as well as in the *target* trials compared with the *early*_25*%*_ trials (*n* = 28, *W* = 48, *p* = 0.001 *FDR corrected*, CI^95^ = [−0.250, −0.082], *rrb* = −0.764) and the *mid*_50*%*_ trials (*n* = 28, *W* = 108, *p* = 0.045 *FDR corrected*, CI^95^ = [−0.175, −0.008], *rrb* = −0.468). The difference between *target* and *late*_75*%*_ trials remained marginal (*n* = 28, *W* = 118, *p* = 0.064 *FDR corrected*, CI^95^ = [−0.143, 0.003], *rrb* = −0.419, *BF*_01_ = 0.855), whereas there was no significant difference between *mid*_50*%*_ and *late*_75*%*_ trials (*n* = 28, *t*(27) = 1.334, *p* = 0.193 *FDR corrected*, CI^95^ = [−0.020, 0.095], *d* = 0.252, *BF*_01_ = 0.928) (Figure 2A).

On the contrary, no significant main effect of the trial group was observed in the *No vision* session (*F*[1.628, 43.960] = 1.785, *p* = 0.185, *η*_*p*_^2^ = 0.062, *BF*_01_ = 2.875) (Figure 2B), indicating the absence of a change in the PSE values as the movement progressed. To compare the PSEs between the *Vision* and *No vision* sessions, we inserted the participants' normalized PSEs in a 4 × 2 repeated measures ANOVA with the trial group and session as within‐subject factors. The ANOVA revealed a significant main effect of the trial group (*F*[1.456, 39.302] = 5.529, *p* = 0.014, *η*_*p*_^2^ = 0.170), no significant main effect of session (*F*[1, 27] = 0.084, *p* = 0.774, *η*_*p*_^2^ = 0.003, *BF*_01_ = 2.586) and a significant trial group × session interaction (*F*[3, 81] = 2.861, *p* = 0.042, *η*_*p*_^2^ = 0.096). However, post hoc comparisons between the PSEs of the same trial group between *Vision* and *No vision* sessions showed no significant differences, with Bayesian analysis providing anecdotal to moderate support for the null hypothesis (*early*_25*%*_ trials: *n* = 28, *t*(27) = 1.774, *p* = 0.349 *FDR corrected*, CI^95^ = [−0.011, 0.150], *d* = 0.335, *BF*_01_ = 1.256; *mid*_50*%*_ trials: *n* = 28, *t*(27) = 0.476, *p* = 0.790 *FDR corrected*, CI^95^ = [−0.053, 0.085], *d* = 0.090, *BF*_01_ = 4.493; *late*_75*%*_ trials: *n* = 28, *t*(27) = −0.270, *p* = 0.790 *FDR corrected*, CI^95^ = [−0.086, 0.066], *d* = −0.051, *BF*_01_ = 4.823; *target* trials: *n* = 28, *t*(27) = −0.871, *p* = 0.783 *FDR corrected*, CI^95^ = [−0.133, 0.054], *d* = −0.165, *BF*_01_ = 3.529). Thus, although we observed a gradual decrease in the PSE values as the movement progressed in the *Vision* session, the overall decrease in the PSEs was comparable between the two sessions.

### 2.3 Reduced Temporal Tuning of Somatosensory Perception When Vision Is Not Available

**FIGURE 3:**
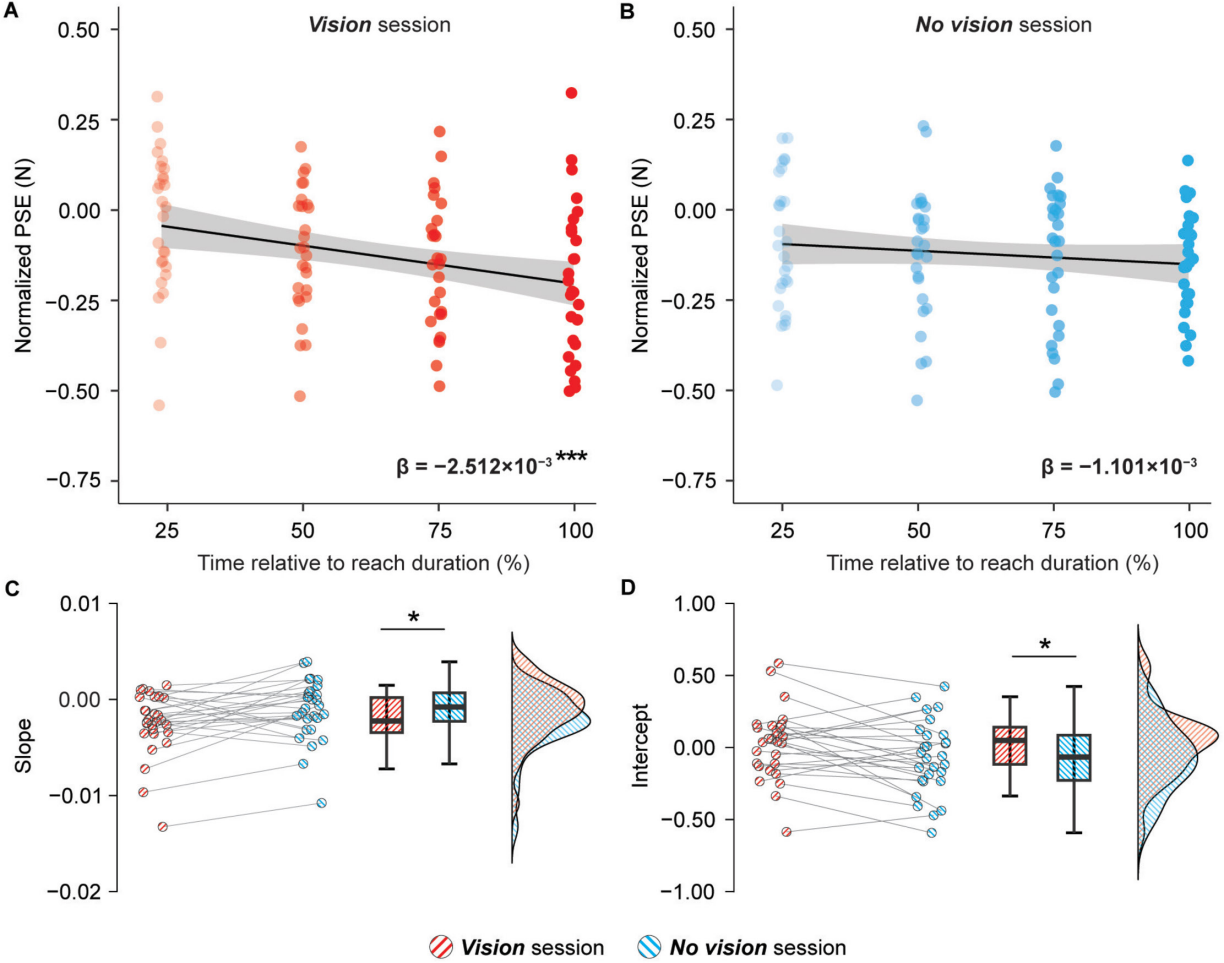
Perceived intensity of the forces applied in *Vision* and *No vision* sessions. (A,B) Baseline‐normalized individual PSE values of every trial group in *Vision* (A) and *No vision* (B) sessions, overlaid with the linear regression model fitted to the group data. Data are jittered (± 1%) to avoid complete overlap, and the shaded area depicts the 95% confidence interval. While individual participant data were fitted with linear models separately, the group‐level fits are shown here for illustrative purposes. The two slope values (*β*) represent the average slopes in *Vision* and *No vision* sessions, and asterisks indicate the significance of slope comparisons against zero (***p < 0.001). One outlier value with very large PSE attenuation in the *target* trials (< −1 N) has been excluded from these panels for clarity. (C) Slopes of the linear regression models fitted to the individual normalized PSE values. In the *Vision* session (red stripes), the slopes were negative and significantly different from zero, whereas the slopes in the *No vision* session (cyan stripes) were not significantly different from zero. The slopes were significantly more negative (i.e., steeper) in the *Vision* session compared to the *No vision* session. (D) Intercepts of the linear regression models fitted to the individual normalized PSE values. The intercepts were significantly more negative in the *No vision* compared to the *Vision* session. (C,D) The markers represent the individual slope and intercept values, boxplots show the medians and interquartile ranges and half‐violin plots illustrate the data distributions as probability densities. Asterisks indicate the significance of statistical comparisons (*p < 0.05).

To further explore the effect of vision on the temporal tuning of the PSEs, we employed a regression analysis treating time as a continuous variable, identically to our previous study (Cemeljic et al. 2025). We used the individual average time points at which the *test* force was delivered in each trial group and modelled the changes in PSEs using a linear regression model (Figure 3A,B). The slopes quantify how PSEs change over the course of the movement, whereas the intercepts reflect the estimated PSEs at the very onset of the movement, thus providing information about the dynamics of somatosensory perception across the movement in the two experimental sessions. This approach allowed us to capture the dynamics of somatosensory perception during movement with greater sensitivity compared to the previous categorical analysis (ANOVA).

As shown in Figure 3A, in the *Vision* session, the extracted slopes were on average negative (*β* = −2.512 × 10^−3^ ± 3.291 × 10^−3^) and significantly different from zero (*n* = 28, *W* = 38, *p* < 0.001, CI^95^ = [−0.003, −0.001], *rrb* = −0.813; *BF*_10_ = 591.287), in line with the ANOVA results and replicating our earlier findings (Cemeljic et al. 2025). The slopes in the *No vision* session were also on average negative (*β* = −1.101 × 10^−3^ ± 3.168 × 10^−3^) as shown in Figure 3B, but not significantly different from zero (*n* = 28, *t*(27) = −1.838, *p* = 0.077, CI^95^ = [−0.001, −0.002], *d* = −0.347), although this was only anecdotally supported by the Bayesian analysis (*BF*_01_ = 1.141). Critically, a direct comparison revealed significantly steeper (more negative) slopes in the *Vision* session than in the *No vision* session (*n* = 28, *t*(27) = −2.556, *p* = 0.017, CI^95^ = [−0.003, −2.787 × 10^−4^], *d* = −0.483) (Figure 3C), showing that the decrease in perceived intensity of the forces during the reaching movement is more pronounced when the visual input was available. In addition, the intercepts in the *Vision* session were significantly higher (i.e., closer to zero) compared with the *No vision* session (*n* = 28, *t*(27) = 2.293, *p* = 0.030, CI^95^ = [0.010, 0.183], *d* = 0.433) (Figure 3D), suggesting that somatosensory perception was less attenuated at the start of the movement when vision was available.

Taken together, the slope and intercept results indicate a distinct modulation pattern of somatosensory perception depending on the availability of vision. In the absence of vision, perception was more uniformly attenuated throughout the movement (i.e., flatter slopes), and this attenuation was already evident at movement onset (i.e., lower intercepts). This reflects a less fine‐tuned, more conservative strategy in the *No vision* session—attenuating sensory input uniformly over time rather than modulating it dynamically. In contrast, the availability of vision fine‐tuned the temporal tuning of somatosensory perception during movement (i.e., less attenuation at the beginning of the movement and greater tuning throughout the movement).

### 2.4 Comparable Task Demands Between *Vision* and *No Vision* Sessions

Where do these differences in the temporal modulation patterns come from? Although our control analysis confirmed that participants performed comparable movements between the two sessions, it could be that participants found the force discrimination task more difficult in the *No vision* session than in the *Vision* session. To test potential differences in task demands, we extracted the just noticeable difference (JND) from the psychometric curves, which represents the precision of force discrimination. When comparing the JNDs between the two groups and across the reaching time, we did not observe any significant differences in the JND values between the trial groups and sessions, with the Bayesian analyses supporting the absence of any effects (all *BF*_01_ > 3) (Text S3 and Figure S5).

### 2.5 Increased Endpoint Variability Relates to Reduced Temporal Tuning of Somatosensory Perception When Vision Is Not Available

Instead, we speculated that the observed differences in the pattern of somatosensory modulation between the *Vision* and *No vision* sessions may stem from less precise predictions about the timing of hands' contact when vision is unavailable. Specifically, in the absence of visual input, the forward model must rely solely on proprioceptive information to update the predictions during the reaching movement. The lack of vision might therefore increase the uncertainty about the positions of both the moving and target hands, and consequently, about the timing of their contact. As a result, this increased uncertainty may lead to reduced temporal tuning of somatosensory perception in the *No vision* session.

**FIGURE 4:**
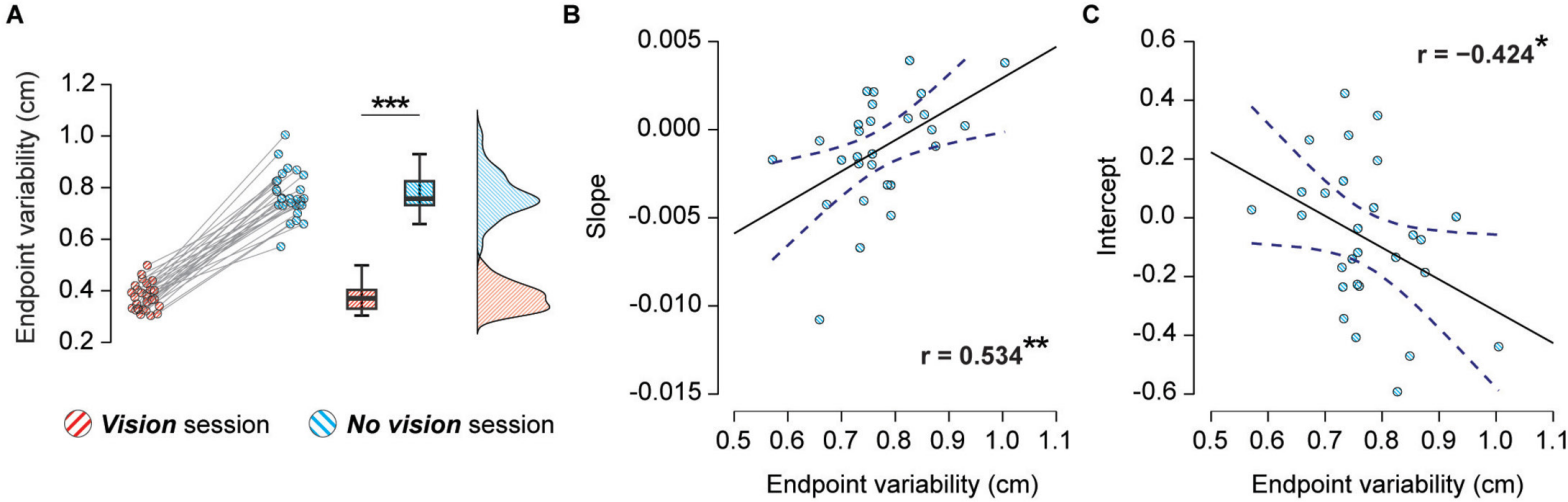
The relationship between reaching endpoint variability and temporal tuning of somatosensory perception. (A) The standard deviations of the 3D positions of the participants' right index finger when tapping the force sensors above their left hand, expressed relative to the initial position at the trial onset. The variability was significantly higher in the *No vision* (cyan stripes) compared to the *Vision* session (red stripes). The markers represent the individual endpoint variability, boxplots show the medians and interquartile ranges, and half‐violin plots depict the data distributions as probability densities. Asterisks depict the significance of statistical comparisons (*** *p* < 0.001). (B,C) Correlation between the variability of the 3D position and the slope (B) and the intercept (C) from the linear regression models in the *No vision* session. Greater variability led to significantly flatter slopes and lower intercepts. The *r* value represents Pearson's correlation coefficient, and asterisks represent the correlation significance (**p < 0.001, *p < 0.05). The area between the dashed lines represents the 95% confidence interval.

To explore this hypothesis, we first examined the participants' variability in localizing their right and left hands, defined as the standard deviation of the 3D position of their right index finger when tapping the force sensors above their left hand (i.e., movement endpoints) (Figure S6). Specifically, for each participant, we calculated the variability when reaching to the left index and ring finger separately and then averaged it across fingers. The variability was significantly higher in the *No vision* session compared to the *Vision* session (*n* = 28, *t*(27) = 22.169, *p* < 0.001, CI^95^ = [0.360, 0.433], *d* = 4.190) (Figure 4A). Together with our previous analysis, this shows that despite matched movement velocity and duration across sessions, the lack of visual input increased the endpoint variability, likely indicating a less precise estimation of the movement goal (i.e., the left finger) or the position of the effector (i.e., the right finger).

Next, we investigated whether this increased variability was related to the reduced temporal tuning of somatosensory perception in the *No vision* session (i.e., flatter slopes and lower intercepts). Specifically, we tested Pearson's correlations between the endpoint variability and the slopes and intercepts of the linear regression models in the *No vision* session. To isolate the variability specifically related to the lack of visual input and thus ensure that any observed correlation was not simply driven by participants' general movement (im)precision, we included the endpoint variability from the *Vision* session as a covariate (i.e., we partialled it out). Indeed, greater variability was significantly associated with flatter slopes (*r*(27) = 0.534, *p* = 0.004; Figure 4B) and lower intercepts (*r*(27) = −0.424, *p* = 0.028; Figure 4C). That is, greater uncertainty in reach goal and/or effector localization without vision led to less precise modulation of somatosensory attenuation during movements to self‐touch. These relationships were not found when performing correlations between the endpoint variability and the slopes (*τ* = 0.069, *p* = 0.624, *BF*_01_ = 3.622) or intercepts (*r*(27) = 0.341, *p* = 0.076, *BF*_01_ = 0.954) in the *Vision* session, possibly due to a substantially decreased endpoint variability when visual feedback was available (Figure 4A).

## 3 Discussion

The present study investigated the influence of vision on the temporal tuning of somatosensory perception during movements to self‐touch. We probed somatosensory perception on the left hand, which served as a target of the reaching movements executed with the right hand, under conditions with and without visual input. Replicating our previous findings (Cemeljic et al. 2025), we observed that participants gradually attenuated the perceived intensity of the forces applied to their left hand during visually guided movements to self‐touch. Importantly, the present results extend these earlier findings by demonstrating that such gradual temporal tuning of somatosensory perception was significantly reduced when participants performed the same task in the absence of vision. Specifically, without visual input, somatosensory perception remained more uniformly attenuated throughout the movement, without exhibiting a significant temporal modulation.

It is worth considering that performing the movements while blindfolded can introduce a higher cognitive and/or attentional load compared to visually guided movements (Giudice 2018; Pigeon et al. 2019). In principle, this can draw the attentional resources away from attending to the tactile stimuli. However, it is unlikely that such hypothesized effects can account for the observed differences in the temporal pattern of somatosensory modulation between the experimental sessions. If there were a higher cognitive and/or attentional load in the *No vision* session compared with the *Vision* session, we would expect worse force discrimination ability (i.e., higher JNDs) in the *No vision* session compared with the *Vision* session. However, we did not observe any significant differences in the JND values between the *Vision* and *No vision* sessions, and this absence of effects was clearly supported by the Bayesian analysis (Figure S5 and Text S3), suggesting comparable force discrimination capacity with and without the visual input.

Rather, the difference in the temporal modulation of somatosensory perception between the two experimental sessions can be explained within the framework of internal forward models. During the reaching movement, the forward model continuously updates its predictions by integrating the available sensory feedback (Brenner and Smeets 2018; Shadmehr et al. 2010; Shadmehr and Krakauer 2008). In the *Vision* condition, both visual and proprioceptive inputs are available, allowing the forward model to refine its predictions about the location of the two hands and the timing of self‐touch as the movement unfolds. As a result, predictions become increasingly precise in the later stages of the movement, reflecting a stronger expectation about the self‐touch around the moment of contact between the hands, and producing a corresponding increase in somatosensory attenuation (Cemeljic et al. 2025). However, when the visual input is removed, the forward model can only rely on proprioceptive feedback. We propose that the lack of visual input contributed to an increased sensory uncertainty about the hands' locations, which reduces the ability of the forward model to sharpen its predictions over time, leading to a flatter temporal profile of somatosensory attenuation in the *No vision* condition. That is, in the absence of visual input, the probability distribution of the self‐touch timing remains wider as the movement progresses, reflecting greater temporal uncertainty. Thus, it fails to support the gradual tuning of somatosensory perception observed when vision is available.

This interpretation aligns with the proposal that information from different sensory modalities is first integrated according to multisensory integration principles and then combined with predictions generated by the forward model (Camponogara 2023; Diedrichsen et al. 2010; Gori et al. 2012; Wolpert et al. 2011). In this way, the forward model can incorporate both visual and proprioceptive inputs to update its predictions, ensuring optimal motor control. It has been proposed that the integration of prior predictions and multisensory feedback is achieved through a Bayesian inference process (Körding and Wolpert 2004; McNamee and Wolpert 2019), in which predictive and sensory signals are weighted according to their relative reliability to optimize the updating of predictions. These accounts are closely related to predictive coding theory (Friston 2005; Rao and Ballard 1999), which posits that the brain continuously generates predictions (‘priors’) across multiple hierarchical levels and updates them based on incoming sensory evidence in a Bayesian manner. From this perspective, the predictions generated by forward models can be viewed as ‘low‐level’ sensory priors within a broader predictive hierarchy in the brain (Ford and Mathalon 2019; Haarsma et al. 2022; Vallortigara 2021).

Our interpretation is additionally supported by the fact that the endpoint variability was greater in the *No vision* session compared with the *Vision* session and that increased endpoint variability was associated with reduced temporal tuning of somatosensory perception during movements to self‐touch performed without vision. Given that the duration, velocity and force of the reaching movements did not differ between the *Vision* and *No vision* sessions, with clear support from the Bayesian analysis (Text S1 and Figure S1), the increased endpoint variability observed without visual input in our experimental design is unlikely to reflect the differences in the motor execution between the sessions (Phataraphruk et al. 2022; Van Beers et al. 2004). Rather, our findings are in line with earlier accounts suggesting that increased endpoint variability reflects higher uncertainty of the forward model (Berret et al. 2021; Crevecoeur et al. 2010; Kessler et al. 2024; Ma‐Wyatt and McKee 2006)—for example, when it has to rely on noisy sensory feedback to update its predictions (Ma‐Wyatt and McKee 2006) or when exposed to novel movement dynamics (Crevecoeur et al. 2010). More specifically, the increased endpoint variability in the *No vision* session may stem from the model's noisy estimations of the position of the effector (here, the right hand) and target (here, the left hand) due to the absence of visual feedback. Indeed, earlier studies have provided ample evidence that visual uncertainty about the position of the target (Amann et al. 2024; Ma‐Wyatt and McKee 2006; McIntyre et al. 1997) and the effector (Desmurget et al. 1995; Rossetti et al. 1995; Tremblay et al. 2017; Vindras et al. 1998) greatly contributes to the increased endpoint variability. This position uncertainty produces temporal uncertainty about the expected moment of contact between the hands, which in turn may limit the brain's ability to fine‐tune somatosensory attenuation in anticipation of touch, leading to the observed reduction in temporal tuning. Our finding that greater endpoint variability was associated with less finely tuned somatosensory perception (i.e., flatter slopes and lower intercepts) in the *No vision* session strongly aligns with this interpretation.

Although vision provided rich sensory feedback to the forward model (Miall et al. 2018; Taylor et al. 2014), such as information about the effector's position relative to the target (Sarlegna and Sainburg 2009; Scheidt et al. 2005), it is not strictly necessary for generating predictive motor control. Humans routinely make accurate predictions based solely on proprioceptive input, as demonstrated by our everyday actions (e.g., reaching to touch our back). Moreover, individuals can adapt to novel motor dynamics and learn new motor tasks even in the absence of visual feedback (Cates and Gordon 2024; Franklin et al. 2007). Our findings are consistent with this capacity: the overall magnitude of somatosensory attenuation did not significantly differ between the *Vision* and *No vision* sessions, suggesting that participants could still generate predictions about the contact of their hands without visual input. However, the absence of temporal modulation in the *No vision* condition indicates that vision plays a critical role in fine‐tuning these predictions over time. Without visual input, the forward model lacks the temporal precision necessary to modulate somatosensory attenuation, resulting in more uniform somatosensory perception throughout the movement.

Under conditions of visual deprivation, such as blindfolding, perception in other sensory modalities can be enhanced due to cross‐modal neuroplasticity in the brain (Merabet et al. 2008; Pagé et al. 2016). Although some studies have reported that blindfolding leads to changes in somatosensory perception—such as improved tactile spatial acuity (Facchini and Aglioti 2003) or altered neural processing of tactile shapes (Weisser et al. 2005)—several others found no such changes (Crabtree and Norman 2014; Radziun et al. 2022; Wong et al. 2011). In line with the latter findings, we did not observe any somatosensory changes indicative of cross‐modal plasticity following blindfolding, as participants' somatosensory precision (JND) did not differ between the *Vision* and *No vision* sessions. It is possible that the relatively short durations of blindfolding, such as in our study (approximately 90 min), are insufficient to reliably induce measurable neuroplastic changes.

Here, we blindfolded sighted participants. However, it remains elusive to what extent permanent visual loss (i.e., blindness) affects the precision of forward model predictions during movements to self‐touch and, in turn, somatosensory perception. Evidence suggests that blind individuals can efficiently adapt to movement changes (DiZio and Lackner 2000) and learn novel motor tasks (Morin‐Parent et al. 2017), indicating that forward models can function efficiently even after loss of vision. Moreover, other studies report a stronger reduction in somatosensory perception on the moving limb in blind participants compared with sighted controls (Casado‐Palacios et al. 2023, 2025). However, it remains unclear whether this reduction reflects alterations in the forward model or is instead driven by other mechanisms, such as weakened interactions between sensory inputs (e.g., proprioceptive and motor cues) resulting from the lack of visual calibration (Gori et al. 2008), which may lead to altered sensory perception (Hötting and Röder 2004; Vercillo et al. 2018). We speculate that during prolonged periods without visual input, such as in blindness, the forward model will adapt to efficiently generate and update predictions based solely on proprioceptive feedback. As a result, blind individuals might exhibit a similar temporal modulation of somatosensory perception during movements to self‐touch, and a comparable magnitude of self‐touch attenuation, to those observed in sighted individuals. Future research should thus aim to clarify how blindness influences somatosensory perception in the context of self‐touch, offering deeper insights into the role of vision in predictive sensorimotor processes.

## 4 Conclusions

The present study demonstrates the crucial role of vision in fine‐tuning the predictions of the internal forward model, and consequently, modulating somatosensory perception during movements to self‐touch. When the forward model can rely on both visual and proprioceptive input, its predictions about the timing of self‐touch become increasingly precise as the movement unfolds, resulting in a gradual attenuation of somatosensory perception. In contrast, when visual input is unavailable and the forward model relies solely on proprioception, its predictions lack temporal precision, and somatosensory perception is less modulated over time. These findings provide novel insights into how vision contributes to self‐touch predictions and modulation of somatosensory perception, advancing our understanding of predictive motor control.

## 5 Materials and Methods

### 5.1 Participants

Thirty‐two naïve participants (17 female, 31 right‐handed and one ambidextrous, aged 18–35 years) were recruited for the study. The sample size was based on our earlier studies using similar tasks and equipment (Cemeljic et al. 2025; Job and Kilteni 2023; Kilteni et al. 2019, 2020, 2021, 2023; Kilteni and Ehrsson 2022, 2024; Timar et al. 2023) and adjusted to ensure a counterbalanced order of experimental sessions and trial blocks. All participants provided informed written consent prior to the start of the study. They reported having normal or corrected‐to‐normal vision, not having any current or previous neurological or psychiatric disorders and not taking psychoactive medication to treat such disorders. The study was approved by the Ethical Review Authority of Stockholm (registration number 2021‐03790 and amendment number 2022‐05686‐02). Participants' handedness was assessed using the Edinburgh Handedness Inventory (median = 100, IQR = 16) (Oldfield 1971). After rejecting invalid trials (see *Trial rejection*), four participants were excluded from further analysis due to poor psychometric fits (McFadden *R* ^2^ < 0.2) in at least one of the trial groups.

### 5.2 Study Pre‐Registration

The hypotheses, experimental methods, and analyses were pre‐registered using the Open Science Framework (https://osf.io/z2wju).

### 5.3 Deviations From Pre‐Registration

We performed three modifications in our analyses from pre‐registration. First, we included repeated measures ANOVA in our statistical analyses to reduce the number of paired comparisons. Second, we performed additional linear regression analysis to further explore the changes in the PSE values over the time course of a reaching movement, identically to our previous study (Cemeljic et al. 2025). Third, to further support the interpretation of the observed somatosensory effects, we tested for correlations between endpoint variability and the slopes and intercepts obtained from the linear regression analysis.

### 5.4 Experimental Setup and Procedures

Participants were seated at a desk, with their left hand resting palm up and their left index and ring fingers placed inside moulded supports (Figure 1A,B). A chin rest (HASOMED Gmbh) and a vacuum pillow (AB Germa) were provided to the participants to minimize their head movements and support their left arm, respectively. A cylindrical probe (diameter: 20 mm) was attached to a lever and placed on top of the pulp of the participants' left index or ring finger (Figure 1A, *probe*). The lever containing the probe was controlled by a DC electric motor (Maxon EC Motor EC 90 flat) and operated by Labjack using custom‐made software written in C++. The probe constantly applied a force of 0.1 N to the participants' left index or ring finger. The applied forces were recorded with a force sensor (FSG15N1A, Honeywell Inc.; diameter, 5 mm; minimum resolution, 0.01 N; response time, 1 ms; measurement range, 0–15 N) positioned inside the probe. Two identical force sensors were put inside two plastic capsules (diameter: 20 mm), which were positioned above the participants' left index and ring fingers while not being in direct contact with the fingers and/or the probe (Figure 1A, *force sensors*). The fourth identical force sensor was placed approximately 25 cm away from the participants' left hand, indicating a starting position of the right hand's reaching movement (Figure 1A, *starting position*). Participants were instructed to apply a constant force on this sensor by continuously pressing it with their right index finger. An elbow pad (Ergorest 3300) supported the participants' right elbow and forearm while simultaneously allowing free movement of their right hand. The position of the participant's right index finger was recorded with a motion tracking sensor (Polhemus Liberty, Micro Sensor 1.8TM) attached to the right index finger with medical tape (Figure 1A, *motion sensor*). Participants wore a pair of headphones through which the reaching instructions, GO‐cues, and the white noise (masking any sounds produced by the motor) were played.

The experimental design was adapted from Cemeljic et al. (2025), with the main difference being the manipulation of the visual feedback (*Vision* and *No vision* sessions). Participants performed the same task in *Vision* and *No vision* sessions, except for being blindfolded for the whole duration of the *No vision* session (Figure 1B, *blindfold*). At the start of every trial, the participants heard the word ‘index’ or ‘ring’, which instructed them which finger of their left hand they were required to reach towards. Immediately after the voice, participants heard an auditory GO‐cue (beep sound of 100 Hz lasting for 100 ms), which prompted them to release the force sensor at the starting position and start the reaching movement with their right hand towards the instructed finger of the left hand. They were instructed to perform a continuous natural movement and to conclude it with a brief tap on the force sensor above the instructed finger (see Figure S7 for an example of the tap force profile). After the tap, participants had to keep their right index finger in the air above the force sensor until receiving the *comparison* force (see *Force trials*) and then return to the starting position. In the *Vision* session, the participants were instructed to focus on the cross placed approximately 12 cm above their left hand (Figure 1A, *fixation cross*). In the *No vision* session, participants were blindfolded for the whole duration of the experiment and further instructed to keep their eyes closed beneath the blindfold. The sessions were completed in a counterbalanced order on separate days. Before the start of each experimental session, participants practiced how to perform the movements and give their responses.

### 5.5 Force Trials

In each session, participants completed two blocks of trials in a counterbalanced order—the *baseline* block (56 trials) and the *reaching* block (280 trials)—resulting in 336 trials per session. In every trial, the participants received two forces on their left index finger: the *test* force and the *comparison* force. The *test* force had a fixed intensity of 2 N, while the *comparison* force varied between seven possible intensities (1, 1.5, 1.75, 2, 2.25, 2.5 and 3 N). Each of the possible *comparison* force intensities was tested 8 times in the *baseline* block and 10 times for each of the four types of trials (see below) in the *reaching* block. The two forces had a fixed duration of 100 ms (see Figure S7 for an example of the *test* force profile). Half of the participants always received the forces on their left index finger (in both experimental sessions) and the other half on their left ring finger.

The *reaching* block consisted of four different trial groups, depending on when the *test* force could occur during a specific trial. The *test* force could thus be applied at 25%, 50% or 75% of the movement time of the last five *reaching* trials (*early*_25*%*_, *mid*_50*%*_ and *late*_75*%*_ trials), as well as simultaneously with participants tapping the force sensor above their left index or ring finger (*target* trials). The movement time of each trial was calculated as a time difference between participants lifting their right index finger from the force sensor at the starting position (the first time point during a trial when the registered force fell below 0.2 N) and tapping the force sensor above the instructed finger (the first time point during a trial when the registered force exceeded 0.2 N). The *comparison* force was presented 1200 ms after the *test* force—therefore, after the reaching movement ended. The calculation of the movement times was performed online using a custom‐made algorithm. Likewise, the *test* force in the *target* trials was applied when the force exerted on the sensor above the instructed finger exceeded 0.2 N (system delay ~20 ms). The number of trials was equally divided between *early*_25*%*_, *mid*_50*%*_, *late*_75*%*_ and *target* trials (70 trials per group), and between reaching towards the left index or left ring finger (35 trials per instructed finger per trial group). In other words, in half of the trials in each trial group, the participants were instructed to reach towards the left index finger, and in the other half towards the left ring finger. In the analysis, the trials were rebinned based on the movement duration of each individual trial (see *Kinematic trials—segmentation*). In the *baseline* trials, participants kept their right index finger motionless at the starting position, receiving the *test* force 100 ms after the trial onset and the *comparison* force 1200 ms later. No auditory go‐cues were played during the *baseline* trials.

After receiving both forces in every trial, the participants had to say which of the two forces felt stronger: the first (*test* force) or the second (*comparison* force). They were told not to balance their responses (having an equal number of ‘first’ and ‘second’ responses) and to make their best guess if the two forces felt equally strong. Participants did not receive any feedback about their responses.

### 5.6 Kinematic Analysis—Preprocessing

The 3D position of the participant's right index finger was recorded at a sampling rate of 240 Hz. The force recordings were co‐registered with the kinematic recordings using transistor–transistor logic (TTL) signals, which indicated the trial onset, as well as the onset and offset of the *test* and the *comparison* force. Recorded positions were smoothed using a moving average filter of 5 data points span (≈21 ms). The analysis of kinematic data was performed in MATLAB 2024a (Mathworks Inc 2024).

### 5.7 Kinematic Analysis—Segmentation

For each trial of the reaching block, we calculated the 3D velocity as the first derivative of the recorded positions of the right index finger. Next, we calculated the movement onset based on the velocity of the reaching movement rather than taking the time when the force sensor at the starting position was released, as was done in the online calculation during the experiment. The movement onset in the analysis was thus defined as the first time point when the 3D velocity of the right index finger exceeded 5 cm/s for the next 100 ms, as in our previous study using a similar task (Cemeljic et al. 2025). This kinematic onset occurred (mean ± SD) 22 ± 15 ms and 20 ± 16 ms after the release of the force sensor at the starting position in *Vision* and *No‐vision* sessions, respectively. Movement offset, as during the experiment, was defined as the first time point when the force exerted on the sensor above the left index or ring finger exceeded 0.2 N. Movement duration was defined as the time difference between the movement offset and kinematic movement onset. The peak velocity was defined as the highest velocity value within 67% of the movement duration, enabling us to capture the peak velocity of the reaching movement, typically occurring during the first half of the movement (McCrea et al. 2002), and not the peak velocity associated with the tap on the force sensor at the end of the movement.

When needed, the *early*_25*%*_, *mid*_50*%*_ and *late*_75*%*_ trials were re‐binned based on the movement duration of each specific trial. More specifically, trials were assigned (a) in the *early*_25*%*_ trial group if the *test* force occurred > 12.5% and ≤ 37.5% of the movement duration in that particular trial, (b) in the *mid*_50*%*_ trial group if the *test* force occurred > 37.5% and ≤ 62.5% of the movement duration in that particular trial and (c) in the *late*_75*%*_ trial group if the *test* force occurred > 62.5% and ≤ 87.5% of the movement duration in that particular trial. To ensure the equal size of the three bins, the trials in which the *test* force occurred ≤ 12.5% or > 87.5% of the movement duration did not enter the subsequent analysis (see *Trial rejection*). After the trial rejection, 196 out of 5213 (4%) *early*_25*%*_, *mid*_50*%*_ and *late*_75*%*_ trials were rebinned in the *Vision* session, and 289 out of 4761 (6%) *early*_25*%*_, *mid*_50*%*_ and *late*_75*%*_ trials were rebinned in the *No vision* session.

### 5.8 Trial Rejection

We excluded (a) any trial in which the *test* force was improperly applied (*test* < 1.85 N or *test* > 2.15 N), (b) any *baseline* trial in which the participants started moving with their right index finger, (c) any *reaching* trials in which participants failed to press the force sensors above their left hand, pressed them multiple times or pressed both sensors during the same trial, (d) any *reaching* trials in which the participants did not move as instructed, that is, not making the continuous movement, missing the force sensors when pressing, moving during *comparison* force or moving away from their left hand (> 5 cm horizontally) before the *comparison* force was applied and (e) any *early*_25*%*_, *mid*_50*%*_ and *late*_75*%*_ trials in which the *test* force occurred ≤ 12.5% or > 87.5% of the movement duration of a specific trial.

In total, out of 9408 administered trials (28 × 336) per session, we rejected 777 (8%) trials in the *Vision* session and 1405 (15%) trials in the *No vision* session. On average, 56 *baseline*, 63 *early*_25*%*_, 66 *mid*_50*%*_, 57 *late*_75*%*_ and 66 *target* trials were applied in the *Vision* session, and 56 *baseline*, 59 *early*_25*%*_, 59 *mid*_50*%*_, 52 *late*_75*%*_ and 60 *target* trials in the *No vision* session.

### 5.9 Psychophysical Fits

We fitted the participants' responses with a generalized linear model (Equation 1) using the *glm* function of the *stats* package in R:

(1) $$p=\frac{e^{\beta 0+\beta 1x}}{1+e^{\beta 0+\beta 1x}}$$

From the model, we extracted the point of subjective equality ($\mathrm{PSE}=-\frac{\beta 0}{\beta 1}$), which corresponds to the intensity where the *test* and *comparison* forces feel equally strong (*p* = 0.5). We have also extracted the JND, which corresponds to the difference between the *p* = 0.5 and *p* = 0.75 thresholds and represents the participants' force discrimination capacity ($\mathrm{JND}=\frac{\log\left(3\right)}{\beta 1}$). PSE was our primary variable of interest because it quantifies the perceived intensity of the *test* force, while JND was used in the additional analysis to rule out any differences in the task demands between different experimental sessions and trials (higher JND suggests poorer discrimination ability and thus greater difficulty of the task). The *comparison* forces were re‐binned to the nearest of seven possible force intensities (1, 1.5, 1.75, 2, 2.25, 2.5 or 3 N) before fitting the responses. McFadden's pseudo *R* ^2^ was used to judge the goodness of a psychometric fit.

### 5.10 Statistical Analysis

Statistical analysis was performed in R (version 4.4.0) (R Core Team 2024) and JASP (version 0.18.3) (JASP Team 2024). Data were analysed using repeated‐measures analysis of variance (ANOVA) with Greenhouse–Geisser corrections when the sphericity assumption was violated. For every ANOVA, we report the corresponding *F*‐statistic, *p*‐value and the effect size given by the partial eta‐squared (*η*_*p*_^2^). Paired comparisons were performed with parametric (paired t‐tests) or nonparametric (Wilcoxon signed‐rank) tests depending on the normality of the data distribution, as assessed with the Shapiro–Wilk test. We report the corresponding statistic (*t‐* or *W‐*statistic), 95% confidence interval (CI^95^), and the effect size (Cohen's *d* or rank‐biserial correlation, *rrb*). In the case of multiple comparisons, the *p*‐values were corrected using the false discovery rate (FDR) (Benjamini and Hochberg 1995) and are denoted as *FDR‐corrected* in the manuscript. Linear regression was performed using the *lm* function from the R *stats* package. Bayesian analysis was conducted to provide evidence for the support of the null hypothesis. Bayesian paired comparisons and correlations were performed with JASP's default Cauchy priors of 0.707 and stretched beta priors of 1, respectively, while Bayesian repeated‐measures ANOVA had a fixed seed of 123 to ensure repeatability. We interpreted the Bayes factors *BF*_01_ between 1 to 3 as ‘anecdotal’, between 3 to 10 as ‘moderate’, and above 10 as ‘strong’ support for the null hypothesis (Quintana and Williams 2018; Van Doorn et al. 2021). Depending on whether the data were normally distributed or not, Pearson's *r* and Kendall's *τ* were calculated for the correlation analyses, respectively. We reported mean ± SD for normally distributed variables, and median and IQR for non‐normally distributed variables.

## Author Contributions

**Noa Cemeljic:** conceptualization, data curation, formal analysis, visualization, writing – original draft, writing – review and editing. **Konstantina Kilteni:** conceptualization, funding acquisition, supervision, writing – review and editing.

## Funding

This work was supported by the European Research Council (101039152), Åke Wibergs Foundation (M20‐0038), Karolinska Institutet (2022‐01634) and Swedish Research Council (VR Starting Grant 2019‐01909 and VR Project Grant 2024‐00906).

## Ethics Statement

The study was approved by the Ethical Review Authority of Stockholm (registration number 2021‐03790 and amendment number 2022‐05686‐02).

## Conflicts of Interest

The authors declare no conflicts of interest.

## Supporting information


**Figure S1:** Duration, peak velocity, and force intensity of participants' reaching movements in *Vision* and *No vision* sessions. There were no significant differences in the movement duration (A‐B), peak velocity (C‐D), and the force by which the participants pressed the force sensors above their left hand (E‐F) between the two experimental sessions (Text S1). Bayesian analyses supported the absence of any differences (all *BF*_01_ > 3). The markers represent the individual values, boxplots show the medians and interquartile ranges, and half‐violin plots depict the data distributions as probability densities.
**Figure S2:** Baseline‐normalized PSE group data (mean ± SEM) separated by session, trial group, and congruency. There was no significant main effect of congruency nor interactions between congruency and trial group and/or experimental session, indicating no influence of congruency on the observed PSE values (Text S2).
**Figure S3:** Fitted logistic models of participants' responses in the *Vision* session for each trial group. The *baseline* trials are depicted in black, while the *reaching* trials are depicted in red (*early*_25_ *_%_* = 25% opacity, *mid*_50_ *_%_* = 50% opacity, *late*_75_ *_%_* = 75% opacity, and *target* = 100% opacity).
**Figure S4:** Fitted logistic models of participants' responses in the *No vision* session for each trial group. The *baseline* trials are depicted in black, while the *reaching* trials are depicted in cyan (*early*_25_ *_%_* = 25% opacity, *mid*_50_ *_%_* = 50% opacity, *late*_75_ *_%_* = 75% opacity, and *target* = 100% opacity).
**Figure S5:** Baseline‐normalized JND values in *Vision* and *No vision* sessions. (A‐B) Baseline‐normalized group JND values for every trial group (mean ± SEM) in *Vision* (A) and *No vision* (B) sessions. There were no significant main effects of the trial group and session, nor their interaction, demonstrating comparable task difficulty between different trials and experimental sessions (Text S3).
**Figure S6:** Example of changes in endpoint variability between experimental sessions. 3D endpoint positions of the right finger were less variable in the *Vision* session (A) compared to the *No vision* session (B). Each marker represents the 3D position of the right index finger when pressing above the left index finger in individual trials from one representative participant.
**Figure S7:** Example of a force profile of the *test* force (red) and of the participants' active tap on the force sensor (blue). Both the *test* and the *comparison* force lasted 100 ms, and the participants were instructed to only briefly tap on the force sensor above their left index or ring finger. The figure shows one *target* trial from a representative participant.
**Text S1:** No differences in reaching duration, velocity, and force between the experimental sessions.
**Text S2:** No effects of finger congruency on the PSEs.
**Text S3:** No effects of the trial group and experimental session on JNDs.

**Table S1:** Supporting Information.

## Data Availability

The data that support the findings of this study are available in the Supporting Information of this article.
