# Supplementary material for: Vision Fine‐Tunes Predictions of Bimanual Self‐Touch
Source: Eur J Neurosci. 2026 Feb 22;63(4):e70435. doi: 10.1111/ejn.70435 (PMC12926622; doi:10.1111/ejn.70435)
Supplement: Supplementary file 1 — Figure S1: Duration, peak velocity, and force intensity of participants' reaching movements in Vision and No vision sessions. There were no significant differences in the movement duration (A‐B), peak velocity (C‐D), and the force by which the participants pressed the force sensors above their left hand (E‐F) between the two experimental sessions (Text S1). Bayesian analyses supported the absence of any differences (all BF 01 > 3). The markers represent the individual values, boxplots show the medians and interquartile ranges, and half‐violin plots depict the data distributions as probability densities. Figure S2: Baseline‐normalized PSE group data (mean ± SEM) separated by session, trial group, and congruency. There was no significant main effect of congruency nor interactions between congruency and trial group and/or experimental session, indicating no influence of congruency on the observed PSE values (Text S2). Figure S3: Fitted logistic models of participants' responses in the Vision session for each trial group. The baseline trials are depicted in black, while the reaching trials are depicted in red (early 25 % = 25% opacity, mid 50 % = 50% opacity, late 75 % = 75% opacity, and target = 100% opacity). Figure S4: Fitted logistic models of participants' responses in the No vision session for each trial group. The baseline trials are depicted in black, while the reaching trials are depicted in cyan (early 25 % = 25% opacity, mid 50 % = 50% opacity, late 75 % = 75% opacity, and target = 100% opacity). Figure S5: Baseline‐normalized JND values in Vision and No vision sessions. (A‐B) Baseline‐normalized group JND values for every trial group (mean ± SEM) in Vision (A) and No vision (B) sessions. There were no significant main effects of the trial group and session, nor their interaction, demonstrating comparable task difficulty between different trials and experimental sessions (Text S3). Figure S6: Example of changes in endpoint variability between experimental ses [file EJN-63-0-s002.pdf]

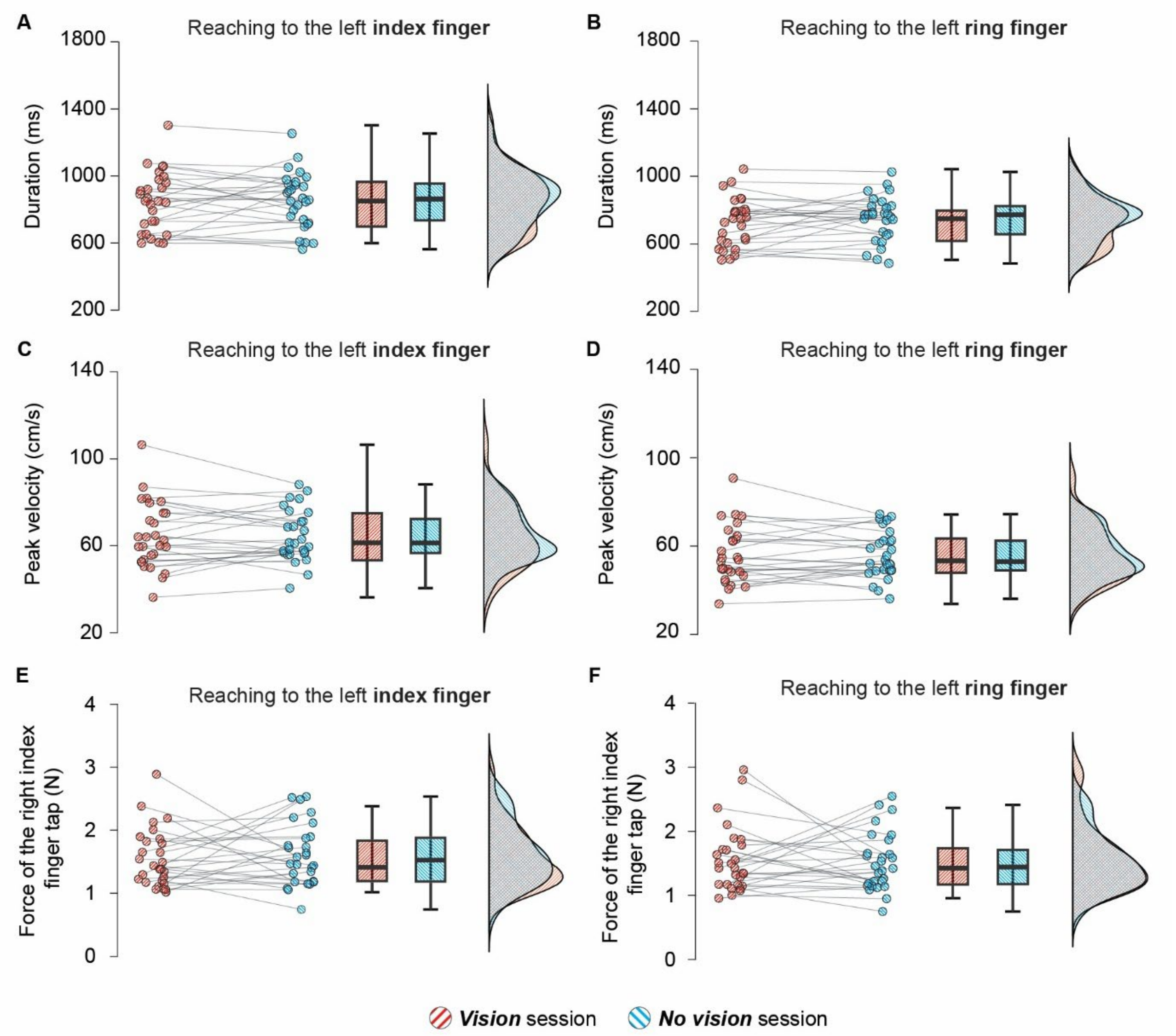

Supplementary Figure S1. Duration, peak velocity, and force intensity of participants' reaching movements in *Vision* and *No Vision* sessions. There were no significant differences in the movement duration (**A-B**), peak velocity (**C-D**), and the force by which the participants pressed the force sensors above their left hand (**E-F**) between the two experimental sessions (**Supplementary Text S1**). Bayesian analyses supported the absence of any differences (all $BF_{01} > 3$). The markers represent the individual values, boxplots show the medians and interquartile ranges, and half-violin plots depict the data distributions as probability densities.

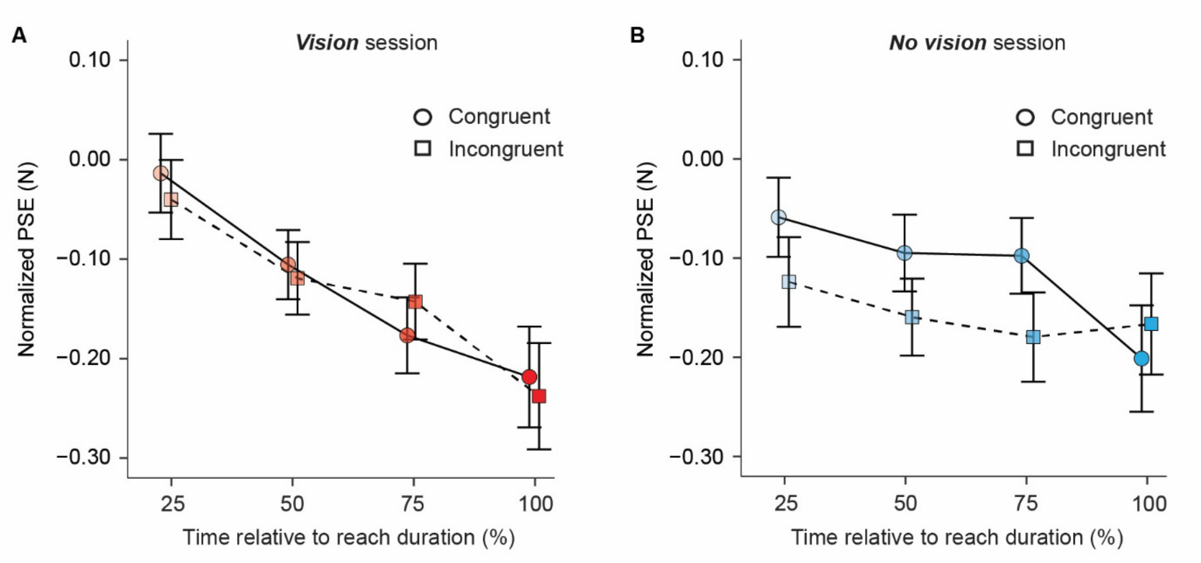

Supplementary Figure S2. Baseline-normalized PSE group data (mean ± SEM) separated by session, trial group, and congruency. There was no significant main effect of congruency nor interactions between congruency and trial group and/or experimental session, indicating no influence of congruency on the observed PSE values (**Supplementary Text S2**).

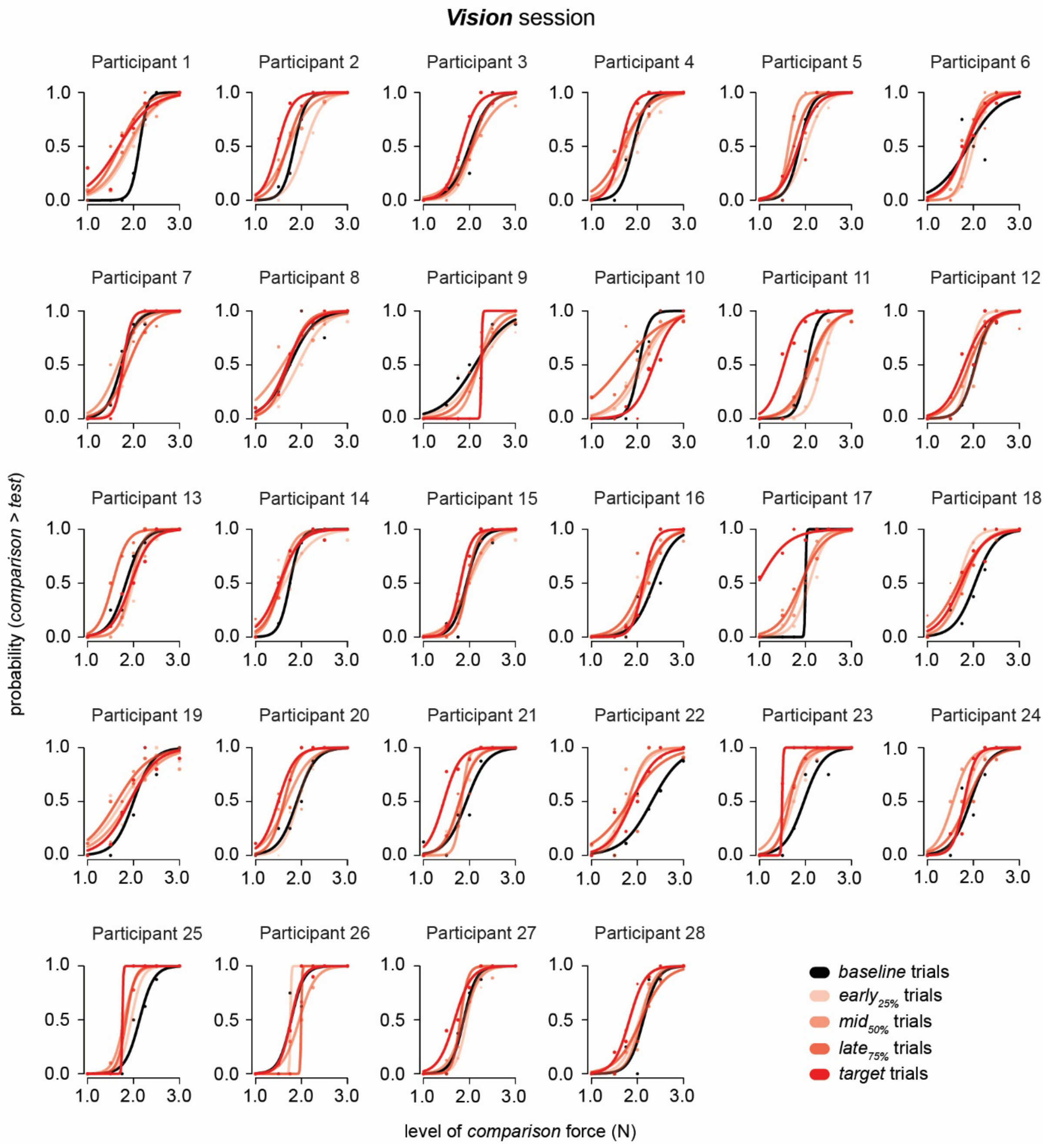

Supplementary Figure S3. Fitted logistic models of participants' responses in the *Vision* session for each trial group. The *baseline* trials are depicted in black, while the *reaching* trials are depicted in red ($early_{25\%}$ = 25% opacity, $mid_{50\%}$ = 50% opacity, $late_{75\%}$ = 75% opacity, and *target* = 100% opacity).

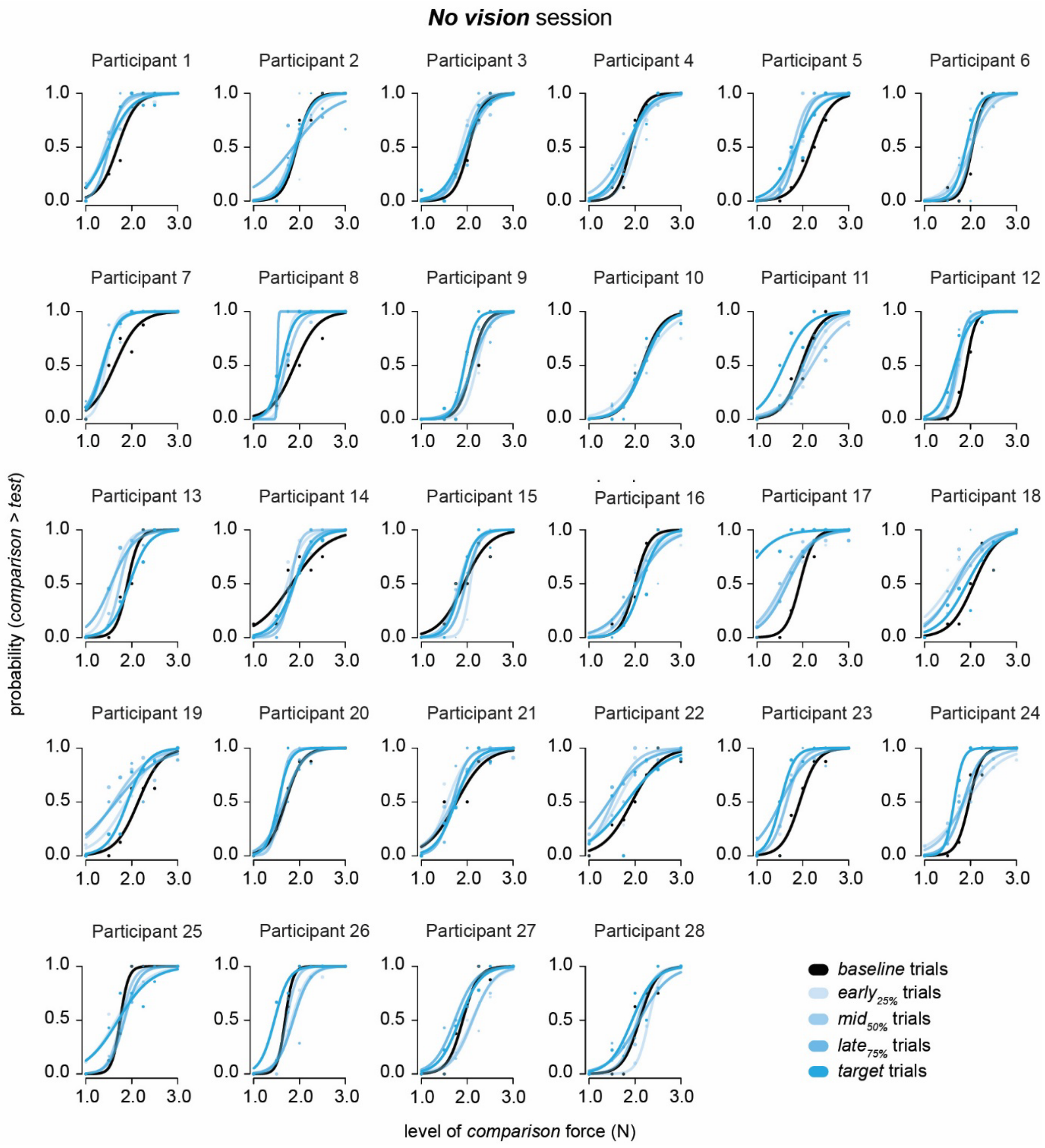

Supplementary Figure S4. Fitted logistic models of participants' responses in the *No vision* session for each trial group. The *baseline* trials are depicted in black, while the *reaching* trials are depicted in cyan ($early_{25\%}$ = 25% opacity, $mid_{50\%}$ = 50% opacity, $late_{75\%}$ = 75% opacity, and *target* = 100% opacity).

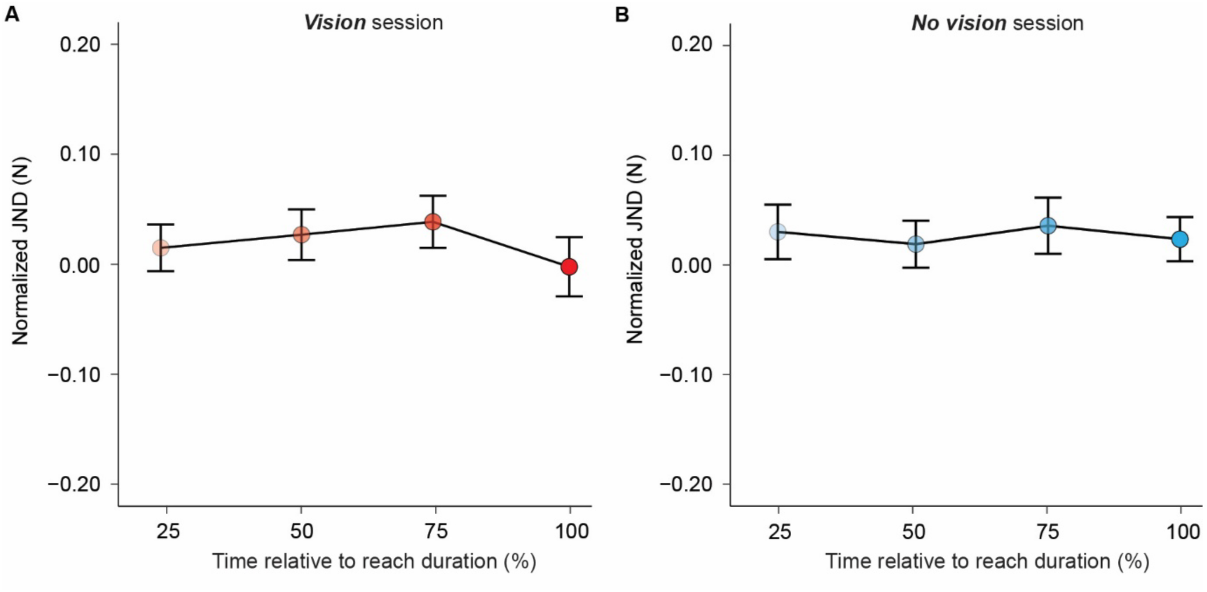

Supplementary Figure S5. Baseline-normalized JND values in *Vision* and *No vision* sessions. (A-B) Baseline-normalized group JND values for every trial group (mean ± SEM) in *Vision* **(A)** and *No vision* **(B)** sessions**.** There were no significant main effects of the trial group and session, nor their interaction, demonstrating comparable task difficulty between different trials and experimental sessions (**Supplementary Text S3**).

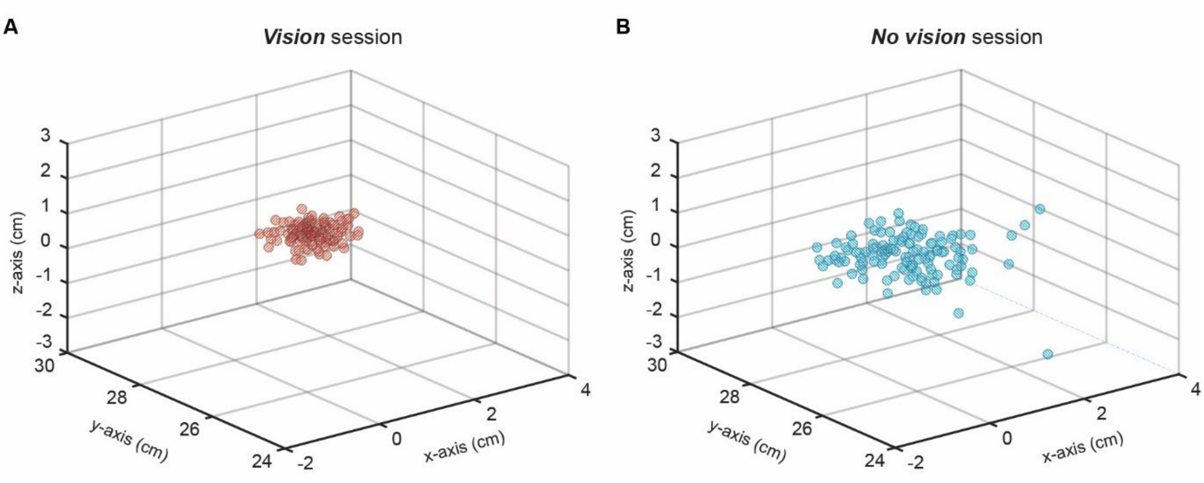

Supplementary Figure S6. Example of changes in endpoint variability between experimental sessions. 3D endpoint positions of the right finger were less variable in the *Vision* session **(A)** compared to the *No Vision* session **(B)**. Each marker represents the 3D position of the right index finger when pressing above the left index finger in individual trials from one representative participant.

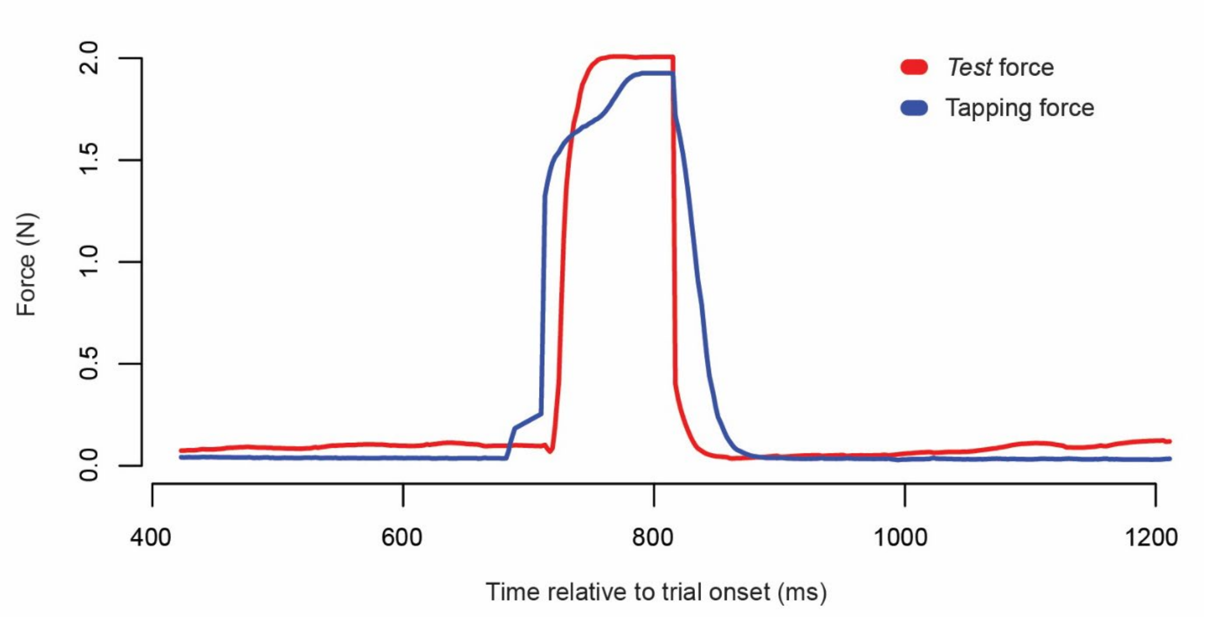

Supplementary Figure S7. Example of a force profile of the *test* force (red) and of the participants' active tap on the force sensor (blue). Both the *test* and the *comparison* force lasted 100 ms, and the participants were instructed to only briefly tap on the force sensor above their left index or ring finger. The figure shows one *target* trial from a representative participant.

Supplementary Text S1. No differences in reaching duration, velocity, and force between the experimental sessions

Participants performed movements of similar durations (mean ± SD) between the sessions when reaching to the left index finger (*Vision*: 846 ± 177 ms, *No vision*: 857 ± 165 ms; $n = 28$, $W = 218$, $p = 0.745$, CI^{95}= [−52.893, 49.866], *rrb* = 0.074; $BF_{01} = 4.907$) (**Supplementary Figure S1A**) and left ring finger (*Vision*: 727 ± 143 ms, *No vision*: 748 ± 134 ms; $n = 28$, $t(27) = -0.979$, $p = 0.336$, CI^{95}= [−62.099, 21.983], $d = -0.185$; $BF_{01} = 3.226$) (**Supplementary Figure S1B**). Furthermore, the peak velocity did not differ between the sessions when reaching to the left index finger (*Vision*: 64 ± 15 cm/s, *No vision*: 65 ± 12 cm/s; $n = 28$, $t(27) = -0.122$, $p = 0.904$, CI^{95}= [−3.606, 3.201], $d = -0.023$; $BF_{01} = 4.953$) (**Supplementary Figure S1C**) and left ring finger (*Vision*: 56 ± 13 cm/s, *No vision*: 56 ± 11 cm/s; $n = 28$, $t(27) = -0.028$, $p = 0.978$, CI^{95}= [−2.892, 2.815], $d = -0.005$; $BF_{01} = 4.986$) (**Supplementary Figure S1D**). Finally, participants applied comparable forces when pressing the force sensor above their left index finger (*Vision*: median (IQR) = 1.41 (0.64) N, *No vision*: 1.61 ± 0.48 N; $n = 28$, $t(27) = 0.510$, $p = 0.614$, CI^{95}= [−0.291, 0.175], $d = -0.096$; $BF_{01} = 4.425$) (**Supplementary Figure S1E**) and left ring finger (*Vision*: median (IQR) = 1.43 (0.57) N, *No vision*: 1.52 ± 0.45 N; $n = 28$, $t(27) = 0.233$, $p = 0.817$, CI^{95}= [−0.214, 0.268], $d = 0.044$; $BF_{01} = 4.864$) (**Supplementary Figure S1F**). Similar results were obtained when comparing the variability (*i.e.*, standard deviation) of these parameters between the *Vision* and *No vision* sessions (all BF_{01} between 2.822 − 4.657), suggesting comparable variability in movement execution between the two sessions.

Supplementary Text S2. No effects of finger congruency on the PSEs

As previously mentioned, participants were instructed to tap above their left index or ring finger with their right index finger. Half of the participants received forces on their left index finger and half on their left ring finger, resulting in 50% congruent trials (*i.e.*, tapping above the left index finger and receiving forces on the left index finger, or tapping above the left ring finger and receiving forces on the left ring finger) and 50% incongruent trials (*i.e.*, tapping above the left index finger and receiving forces on the left ring finger, or vice versa).

A 4×2×2 repeated measures ANOVA on the normalized PSEs ($PSE_{reaching} - PSE_{baseline}$) with the trial group ($early_{25\%}$, $mid_{50\%}$, $late_{75\%}$, *target*), session (*Vision* and *No vision*), and congruency (congruent and incongruent) as within-subjects factors revealed no significant main effect of congruency ($F(1, 27) = 2.477$, $p = 0.127$, $\eta_p^2 = 0.084$) nor session × congruency ($F(1, 27) = 2.149$, $p = 0.154$, $\eta_p^2 = 0.074$), trial group × congruency ($F(3, 81) = 1.138$, $p = 0.339$, $\eta_p^2 = 0.040$) and trial group × session × congruency ($F(3, 81) = 2.003$, $p = 0.120$, $\eta_p^2 = 0.069$) interactions (**Supplementary Figures S2A-S2B**). A Bayesian repeated measures ANOVA using the same factors provided moderate to strong evidence against the main effect of congruency ($BF_{01} = 5.274$), session × congruency ($BF_{01} = 10.298$), trial group × congruency ($BF_{01} = 15.018$), and trial group × session × congruency ($BF_{01} = 33.350$) interactions.

Supplementary Text S3. No effects of the trial group and experimental session on JNDs

A 4×2 repeated measures ANOVA on normalized JND values ($JND_{reaching} - JND_{baseline}$) with trial group and session as within-subject factors revealed no significant main effects of trial group ($F(3, 81) = 1.157$, $p = 0.332$, $\eta_p^2 = 0.041$) and session ($F(1, 27) = 0.074$, $p = 0.788$, $\eta_p^2 = 0.003$) nor trial group × session interaction ($F(3, 81) = 0.713$, $p = 0.547$, $\eta_p^2 = 0.026$) (**Supplementary Figure S5A-S5B**). This was further supported by a Bayesian repeated measures ANOVA, which provided moderate to strong support for the null model for a main effect of trial group ($BF_{01} = 13.041$), main effect of session ($BF_{01} = 3.993$), and trial group × session interaction ($BF_{01} = 35.059$).
